# How do spine surgeons cope with psychological distress: results of a cross-sectional study

**DOI:** 10.1007/s10143-023-02088-z

**Published:** 2023-07-22

**Authors:** Darius Kalasauskas, Malte Ottenhausen, Irene Irene, Andrea Chmitorz, Klaus Lieb, Florian Ringel

**Affiliations:** 1 Department of Neurosurgery, University Medical Centre of the Johannes Gutenberg University Mainz, Mainz, Germany; 2 Faculty of Social Work, Health and Nursing Sciences, Esslingen University of Applied Sciences, Esslingen, Germany; 3 Department of Psychiatry and Psychotherapy, University Medical Centre of the Johannes Gutenberg University Mainz, Mainz, Germany; 4 Leibniz Institute for Resilience Research (LIR), Mainz, Germany

**Keywords:** Burnout, Psychological stress, Resilience, Coping, Surgeons, Quality of life, Survey, Mental health, Working conditions, Depression

## Abstract

Cross Sectional Study/Online Survey. In this study, we sought to assess stress, psychological distress, resilience, and coping strategies among spine surgeons in German-speaking countries. Recent studies have reported high rates of stress and burnout among surgeons. A survey via Survey Monkey™ was conducted among spine surgeons practicing in German-speaking countries using validated questionnaires for perceived stress, mental burden, resilience, and quality of life. Data on working situation and demographics were also collected. 582 surgeons responded to the survey, representing 15% of those surveyed. 79% of respondents were satisfied with their professional success. Mental burden was higher than in the general population, as was perceived stress. Chairpersons were exposed to the lowest levels of perceived stress and mental burden. Mental distress was high (GHQ ≥ 12) in 59% of residents and 27% chairpersons. Self-reported psychological resilience was higher than levels found in the general population and highest among chairpersons. Quality of life was comparable to levels reported in the general population. There were statistically significant correlations between perceived stress and mental burden scores (r _s_ = 0.65, p < 0.001). Career level (senior physicians vs. residents, OR 0.26; 95% CI 0.10-0.66), perceived stress (OR 1.54; 95% CI 1.33-1.77), self-reported resilience (OR 0.53; 95% CI 0.33-0.84), and mental composite score (SOR 0.86; 95% CI 0.83-0.90) were predictors of high mental burden. There was no interaction between perceived stress and resilience on mental burden (p = 0.835). Spine surgeons are exposed to higher levels of stress than the general population, which are associated with higher mental distress. More professional experience and higher levels of psychological resilience are associated with lower levels of stress.

**Supplementary Information:**

The online version contains supplementary material available at 10.1007/s10143-023-02088-z.

## Introduction

Physicians’ mental health and satisfaction depend in large part on their working conditions. A variety of factors that influence physicians well-being have been described: These include external factors related to the work environment, such as career satisfaction, stress and working hours, as well as individual factors such as psychological resilience, coping strategies, physical health and habits [1]. Previous studies have reported an increasing rate of burnout and mental disorders, especially among surgeons, resulting in a reduced quality of life [2, 3]. Although a recent study suggested that physicians have higher levels of resilience than the general working population [4], all medical professions are at increased risk for suicide [5], and the prevalence of suicide among physicians is increasing [6]. Orthopedic surgery is the most commonly reported specialty among surgeons to commit suicide, accounting for nearly 30% of all physician suicides [6].

The amount of stress a physician is able to cope with varies widely and depends on the individual’s psychological resilience. In general, psychological resilience refers to the phenomenon that many people, despite exposure to adversity, adapt to the life’s challenges and maintain mental health. Adversity refers to exposure to macrostressors, such as the loss of a job or the death of a close relative, and microstressors, such as the daily commute to work, time pressures at work, or minor interpersonal conflicts [7]. The concept of psychological resilience was first proposed in the early 1970s. Since then, the definition has changed from a stable personality trait (“a hardy person”) to an outcome of a process or the dynamic process itself [8].

The burnout rate among orthopedic surgeons, trauma surgeons, and neurosurgeons, the three disciplines that perform spine surgery, has been described as exceptionally high, and residents trained in these disciplines are a highly vulnerable group [9]. On the other hand, among medical specialties, surgeons had the highest level of perceived distress, but also the highest level of work ability and the lowest score for depression [10].

In the light of these findings, it should be the goal of the medical community to identify the sources of stress, reduce the amount of stress to a tolerable level, and help physicians to better cope with stress.

The purpose of this study was to assess psychological distress, quality of life and resilience as a basis for developing concepts of stress reduction and stress coping mechanisms.

## Methods

The study was reviewed and approved by the local ethics committee (reference number 2019–14,545).

An online-based cross-sectional survey of spine surgeons was created using the SurveyMonkey™ platform. An invitation to participate in the study with a link to the survey was emailed to members of the two largest spine societies in German-speaking countries, the German Society of Spine Surgery (DWG) (2350 members) and the German Society of Neurosurgery (DGNC) (1529 members). The survey was conducted from July 3^rd^ to August 2^nd^, 2019. As parallel membership in both organizations is possible and some members of the DGNC do not practice spine surgery, the participation rate for the survey could not be calculated. The survey took approximately 20 min to complete and was anonymous.

### Standardized Questionnaires

HRQoL was measured by an 8-item Short Form Health Survey (SF-8). The SF-8 is a short, multi-purpose survey that was shortened from the larger SF-36 survey to reduce respondent burden [11, 12]. In general, mean T-scores of 47 or greater for group-level data are considered average or better in relation to the general population [12].

Mental health was assessed using the General Health Questionnaire (GHQ) [13, 14].

Perceived stress was assessed using a German version of the 4-item Perceived Stress Scale (PSS-4) [15, 16].

Self-reported resilience was measured using a German version of the Brief Resilience Scale (BRS) [17–19].

Self-efficacy refers to the self-assessed confidence in one’s ability to achieve desired outcomes [20] and is an important factor in resilience [21]. The General Self-Efficacy Short Scale (ASKU) was used to measure self-efficacy [22].

### Statistical analysis

Descriptive analysis was used to statistically evaluate the survey data. Differences in means between study subgroups were assessed using one-way ANOVA with Bonferroni correction and t-test, depending on the number of groups. In case of non-normal distribution, Kruskal–Wallis test was used. Chi-squared test was used to evaluate the difference between proportions. Spearman correlation was used to assess associations between the scale scores. A cut-off score of 12 on the GHQ-12 score was used as a marker of significant mental burden [17] for a logistic regression analysis. Statistical analysis was performed using Stata software, version 12, (StataCorp, USA) and SPSS, version 23 (IBM Corporation, USA). P < 0.05 was considered as statistically significant.

## Results

582 respondents completed the survey. The response rate was 15%. The majority of respondents (96%) were from Germany, 98 (17%) were female, the mean age was 45.9 years (range 28–70, SD 9.8). More than 80% (*n* = 475) of the respondents were married, 25% (*n* = 147) had no children, 7% (*n* = 38) had more than 3 children. 30% (*n* = 175) worked in university hospitals, 54% (*n* = 316) in other hospitals and 11% (*n* = 63) in private practice. 67% (*n* = 389) of respondents were neurosurgeons and 32% (*n* = 185) orthopedic and trauma surgeons. There were 12% (*n* = 69) residents, 26% (*n* = 152) senior physicians, 19% (n = 113) department vice-chairs and 23% (*n* = 136) department chairs. Respondents worked a mean of 59 h (range 7–100, SD 12.45) per week. The majority of surgeons (66.7%) performed between 100 and 400 operations per year. 9% (*n* = 53) of respondents performed under 50 operations and 1% (*n* = 7) over 800 operations per year. At least half of the surgeries performed by the majority of respondents were spine cases (Table 1).

**Table 1:** Sample characteristics (*N* = 582)

Total	*N* = 582 (= 100%)		
Gender		Working hours	7 – 100 (56.87 ± 12.45)
Male		Performed operations each year	
Female		< 50	53 (9.1%)
Age	28–70 years (45.9 ± 9.8)	50–100	83 (14.3%)
Family Status		100–200	171 (29.4%)
Married	475 (81.6%)	200–400	223 (38.3%)
Divorced	38 (6.5%)	400–800	45 (7.7%)
Single	65 (11.2%)	> 800	7 (1.2%)
Widowed	4 (0.7%)	Percentage of spine surgery	
Children		0%	10 (1.7%)
0	147 (25.3%)	1–25%	86 (14.8%)
1	85 (14.6%)	25–50%	100 (17.2%)
2	217 (37.3%)	50–75%	120 (20.6%)
3	95 (16.3%)	75–99%	120 (20.6%)
> 3	38 (6.5%)	100%	146 (25.1%)
Specialty		Country of employment	
Orthopedics/Trauma	185 (31.8%)	Germany	557 (95.7%)
Neurosurgery	389 (66.8%)	Switzerland	6 (1%)
Other	8 (1.4%)	Austria	8 (1.4%)
Type of employer		Other	11 (1.9%)
Universityhospital	175 (30.1%)	Satisfaction with professional success	
Hospital	316 (54.3%)	Yes	461 (79.2%)
Private practice	63 (10.8%)	No	121 (20.8%)
Industry	1 (0.2%)	Career opportunities	
Other	27 (4.6%)	Very good	113 (19.4%)
Finished Residency		Good	198 (34%)
Yes	509 (87.5%)	Medium	166 (28.5%)
No	73 (12.5%)	Not that good	72 (12.4%)
Career level		Bad	33 (5.7%)
Chairmen	136 (23.4%)		
Vice Chairmen	113 (19.4%)		
Senior Physician	152 (26.1%)		
Senior Physician	22 (3.8%)		
Board certified	90 (15.5%)		
Resident	69 (11.9%)		

Seventy-nine percent (n = 461) of respondents were satisfied with their professional success, and the majority were optimistic about their career opportunities. Detailed sample characteristics are shown in Table 1. Seven percent (n = 39) of respondents had a diagnosis of a mental illness, including 24 cases of depression. 12% (n = 71) were smokers, 10% (n = 53) of all respondents consumed alcohol five or more days per week. Six percent (n = 33) used prescription and illicit drugs without a medical indication. Antidepressants were the most commonly used drugs with a medical indication (3%, n = 15).

### Perceived stress

**Fig. 1:**
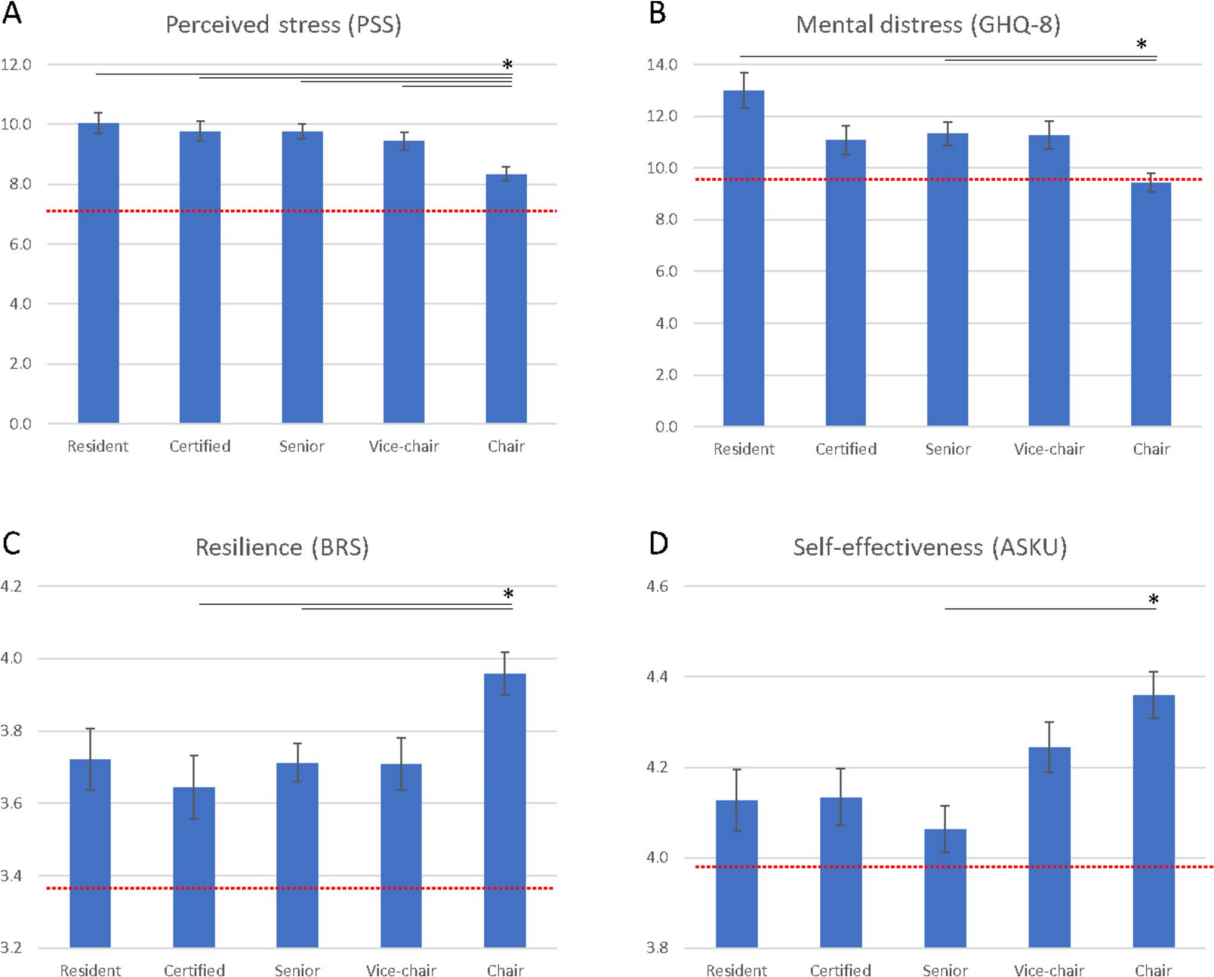
Indicators of stress and resilience among occupational categories of spine surgeons. **A** – The level of perceived psychological stress according to 4-item Perceived stress scale. **B** – The level of mental distress according to 12-item General Health Questionnaire. **C** – The level of resilience according to Brief Resilience Scale. **D** – The level of self-effectiveness among spine surgeons according to ASKU questionnaire. Asterisk marks statistically significant difference (One-way ANOVA, Bonferroni post-hoc test, *p* < 0.05). Red line represents the mean value of representative studies of German population [23]

The level of perceived stress as assessed by the PSS-4 was high among spine surgeons (mean 9.4, SD 2.7) and was higher than the values previously reported for the general population (mean 7.0, SD 2.8, *n* = 1,128) [18]. Among the career level subgroups, perceived stress was significantly lower among chairpersons (mean 8.3, SD 2.5), compared to other positions (vice-chairpersons mean 9.4, SD 3.0, p = 0.047; senior physicians mean 9.8, SD 2.7, *p* = 0.001; board-certified physicians mean 9.8, SD 2.8, *p* = 0.005; residents mean 10.1, SD 2.5, *p* = 0.002) (Fig. 1A).

### Mental burden

A similar trend was observed for mental burden. The mean level of mental burden according to the results of the GHQ-12 questionnaire was 11.0 ± 5.2, which was high compared to the levels found earlier in general population (mean 9.7, SD 4.5, *N* = 1977) [24]. Chairpersons had significantly lower levels of mental burden (mean 9.4, SD 4.2) compared to residents (mean 13.0, SD 5.4, p < 0.001) and senior surgeons (mean 11.3, SD 5.4, *p* = 0.03) (Fig. 1B). The difference between chairpersons and vice-chairpersons (mean 11.3, SD 5.7, *p* = 0.07) and board-certified physicians (mean 11.1, SD 5.1, *p* = 0.20) did not reach statistical significance. In our sample, the number of respondents scoring 12 or more on the GHQ-12 was n = 204 (35%), demonstrating that a significant proportion of spine surgeons suffer from mental burden.

### Resilience

We have found that the psychological resilience, as measured by the BRS, was high in spine surgeons (mean 3.8, SD 0.7). The mean score was higher than that reported in the general population (mean 3.4, SD 1.0, *n* = 1128) [18]. Self-reported BRS-scores were highest among chairpersons (mean 4.0, SD 0.7), which was significantly higher than among board-certified surgeons (mean 3.6, SD 0.8, p = 0.02) and senior physicians (mean 3.7, SD 0.6, *p* = 0.04) but did not reach significance compared to vice-chairpersons (mean 3.7, SD 0.7, *p* = 0.07) and residents (mean 3.7, SD 0.7, *p* = 0.24) (Fig. 1C).

### Self-effectiveness

The level of self-effectiveness, as measured by the ASKU was in mean 4.2, SD 0.6 and was similar to the representative data of the general population in the literature (mean 4.0, SD 0.7, *n* = 1128) [18]. Self-effectiveness was also highest among chairpersons (mean 4.4, SD 0.5), but only reached statistical significance compared to senior physicians (mean 4.1, SD 0.6, *p* = 0.001) (Fig. 1D).

**Fig. 2:**
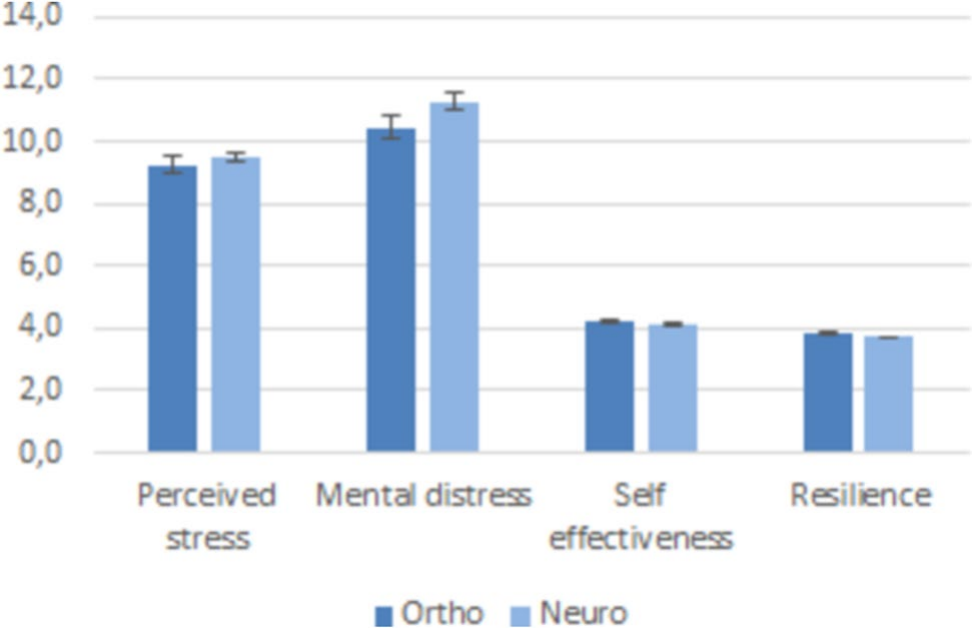
Comparison of Perceived stress, Mental distress, Self-effectiveness and Resilience between orthopaedic-surgeons and neurosurgeons (NS, Student t-test)

**Fig. 3:**
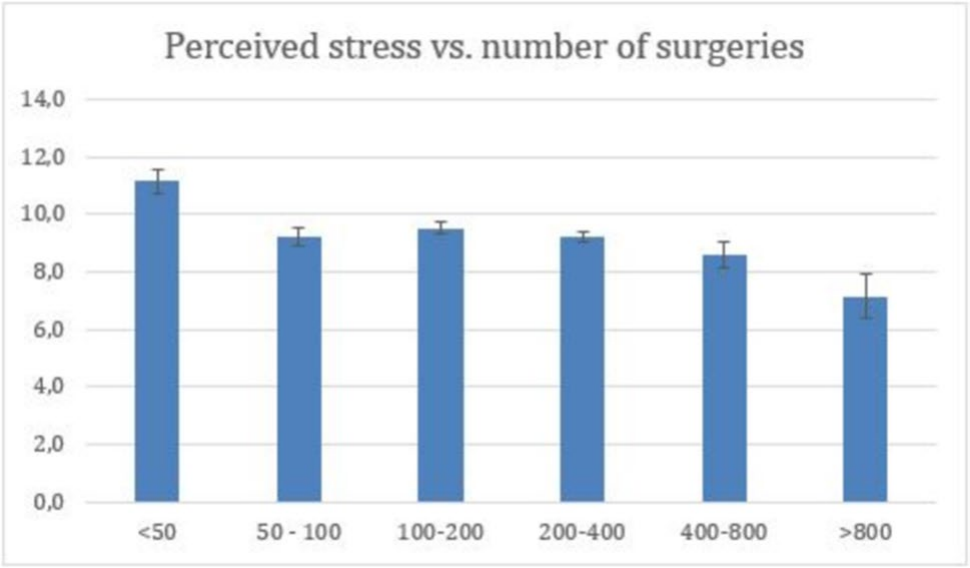
The association between the level of perceived stress, as measured by Perceived Stress Scale and the number of surgeries (*p* < 0.001, Kruskal-Wallis Test)

There was no significant difference in perceived stress, mental burden, self-efficacy and self-reported resilience scores between neurosurgeons and orthopedic/trauma surgeons (Fig. 2). However, there was a marked decrease in perceived stress with an increasing number of surgical procedures per year (*p* < 0.001, Fig. 3). On the other hand, no correlation was found between perceived stress and the number of hours worked per week (r_s_ = 0.06, *p* = 0.15).

### Quality of Life

**Fig. 4:**
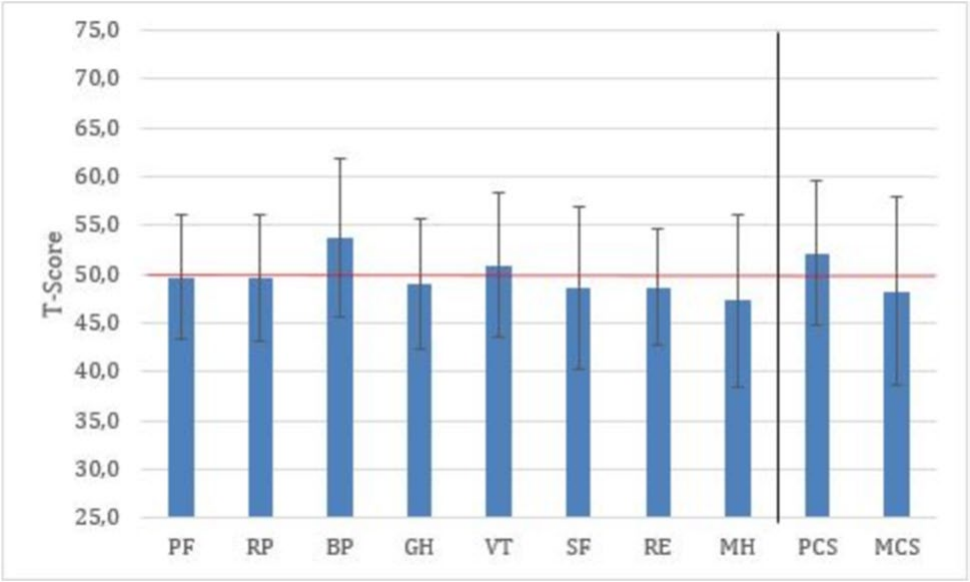
T-Scores of SF-8 health domains as well as composite scores. Error bars represent standard deviation. Physical Functioning (PF), Role-Physical (RP), Bodily Pain (BP), General Health (GH), Vitality (VT), Social Functioning (SF), Role-Emotional (RE) and Mental Health (MH), Physical Component Summary (PCS) and Mental Component Summary (MCS)

The overall quality of life of spine surgeons was similar to that of the general population in all categories (Fig. 4). However, when comparing different career levels, we found that there was a significant difference in the mental component score between chairpersons and residents (mean 51.4, SD 8.4 vs. mean 44.2, SD9.9, *p* < 0.001), vice-chairpersons (mean 47.5, SD 10.1, p = 0.02) as well as senior physicians (mean 47.5, SD 9.4, *p* = 0.01). There was no difference in the physical component score between the groups. The mean scores of all four domains, comprising the mental component were significantly lower among residents. From the domains of physical component, vitality was significantly lower among residents (mean 47.7, SD 7.9) and senior physicians (mean 49.6, SD 7.6) compared to chairpersons (mean 53.7, SD 6.7, p < 0.001 for both comparisons), with the scores of other components being comparable. There was no statistically significant difference in health domains between orthopedic/trauma surgeons and neurosurgeons.

**Fig. 5:**
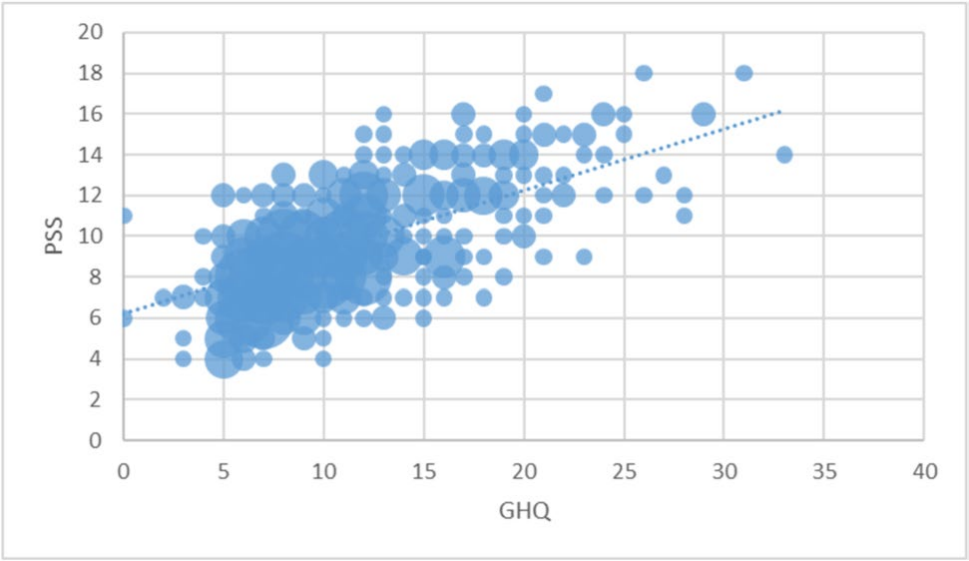
Association between perceived stress, as measured by Perceived Stress Scale and mental distress, as measured by General Health Questionnaire (rs = .65, *p* < 0.001)

There was a positive correlation between PSS and GHQ scores (r_s_ = 0.65, *p* < 0.001), and a significant negative correlation between MCS and PSS (r_s_ = -0.54, *p* < 0.001) and GHQ (r_s_ = -0.73, *p* < 0.001) (Fig. 5), indicating that higher levels of perceived stress and mental burden are associated with lower quality of life.

In a univariate analysis, age, number of surgeries per year, mental illness, being a resident physician versus chairperson, board-certified physician and senior physician, number of hours worked, MCS, PCS, BRS, ASKU and PSS scores were associated with higher mental burden (Table 2). In a multivariate model, being a resident versus being a senior physician, BRS, PSS and MCS remained statistically significant predictors of significant mental burden (last step of multivariate regression is shown in Table 3). Senior physicians were almost four-times less likely than residents to report high levels of mental burden than residents (odds ratio [OR] 0.26; 95% confidence interval [CI] 0.10–0.66). Each additional unit on the PSS increased the odds of mental burden by 54% (OR 1.54; 95% CI 1.33–1.77). The odds of high mental burden decreased by 14% with each unit on the MCS (OR 0.86; 95% CI 0.83–0.90). On the other hand, each additional unit on the BRS (i.e., increasing ability to recover from stress) decreased the odds of mental burden by 47% (OR 0.53; 95% CI 0.33–0.84). There was no statistically significant interaction between PSS and BRS scores on mental burden observed (p = 0.835).

**Table 2:** Univariate logistic regression for significant mental dysfunctions (GHQ12 >  = 12) among spine surgeons. OR – odds ratio, CI – confidence interval

Factor	OR (95% CI)	*p*-value
Gender (male vs. female)	1.40 (0.89–2.21)	
Age (per year)*	0.96 (0.95–0.98)	< 0.001
Number of surgeries/year (per category, as in Fig. 3)*	0.68 (0.58–0.80)	< 0.001
Workplace		
(private vs. university hospital private vs. non-university hospital)	1.03 (0.56–1.89) 1.05 (0.59–1.84)	
Orthopedic vs. Neurosurgery	1.36 (0.93–1.99)	
Family status (single vs. married/partner) †*	3.28 (1.89–5.69)	< 0.001
Diagnosed mental illness* (yes vs. no?)	2.75 (1.34–5.66)	0.006
Job category		
(chairman vs. resident)* (senior physician vs. resident)* (certified physician vs. resident)*	0.26 (0.14–0.49) 0.37 (0.20–0.67) 0.41 (0.21–0.79)	< 0.001 0.001 0.008
Number of working hours (per week?)*	1.02 (1.004–1.03)	0.014
SF-8 Mental Composite score (per 1 point)*	0.82 (0.80–0.85)	< 0.001
SF-8 Physical Composite score (per 1 point)*	0.92 (0.90–0.95)	< 0.001
BRS Score (per 1 point)*	0.24 (0.17–0.33)	< 0.001
ASKU Score (per 1 point)*	0.31 (0.21–0.45)	< 0.001
PSS Score (per 1 point)*	1.82 (1.63–2.04)	0.001

^†^ “single” does not include divorced individuals

**Table 3:** Multivariate logistic regression analysis for significant mental distress (GHQ12 >  = 12). OR – odds ratio, CI – confidence interval

Factor*	OR (95% CI)	*p*-value
Job category		
(chairman vs. resident) (senior physician vs. resident) (certified physician vs. resident)	0.58 (0.22–1.53) 0.26 (0.10–0.66) 0.50 (0.18–1.38)	0.272 < 0.001 0.181
SF-8 Composite Mental score	0.86 (0.83–0.90)	< 0.001
SF-8 Composite Physical score	0.97 (0.93–1.01)	0.098
BRS Score	0.53 (0.33–0.84)	0.006
PSS Score	1.54 (1.33–1.77)	< 0.001

^*^a backward conditional method was used for the factor selection. The factors that were statistically significant in a univariate analysis (i.e. age, number of surgeries, family status, career level, number of working hours, SF-8 composite mental and physical scores, BRS, ASKU and PSS scores) were used for a multivariate model. The table represents values in the final (7^th^) step of backward conditional logistic regression, Nagelkerke R Square 0.621

## Discussion

According to the survey data, the levels of perceived stress and mental burden were high among spine surgeons. However, levels of resilience, i.e. the ability to recover quickly from stressful events, were higher than in the general population. Overall quality of life was comparable to the general population across all domains and there were no differences between orthopedic surgeons, trauma surgeons and neurosurgeons. High level of mental burden was associated with younger age, longer working hours, higher perceived stress, and less experience, among other factors. It was inversely associated with high resilience. In a multivariate analysis, only high resilience, high perceived stress, lower experience, and the composite mental score of the SF-36 remained significant factors for mental burden. Although significant levels of stress were reported, approximately 80% of respondents were satisfied with their career choice.

Job satisfaction and distress are highly dependent on the work setting. The high rate of satisfaction (79%) of the present study population is consistent with data from an American survey of surgeons working in academic and private settings [25]. On the other hand, another study reported that German physician respondents were less satisfied overall than their US counterparts [26] and colleagues from other countries. Because spine surgeons in German speaking countries represent a mixed population of orthopedic surgeons, trauma surgeons, and neurosurgeons, there may be differences between specialties due to different backgrounds. However, no statistically significant differences in stress levels, resilience, and quality of life were found between specialities in our study. However, there is a reason to believe that individual stress levels of surgeons working in high volume centers may be higher than those of surgeons operating on elective cases only.

There was a higher proportion of chairpersons than other positions among the survey respondents. Ninety-four respondents identified themselves as chairperson of a non-university hospital. In addition to possible response bias, the high number may be due to a significant amount of small hospital-affiliated orthopedic departments or private practices specializing in spine surgery. In addition, more experienced surgeons tend to be more active in professional societies, which may explain the relative underrepresentation of residents in the survey.

### High perceived stress is associated with high mental burden and lower quality of life among spine surgeons

This study demonstrated that spine surgeons are exposed to significant amounts of stress, as perceived stress scores were higher than in the general population. In addition, 35% of respondents suffered from significant mental burden. In general, mental burden is high among healthcare professionals, especially in surgical specialties, and can lead to a burnout syndrome [10, 24]. Clinicians exposed to stress are at increased risk of providing suboptimal quality of care to their patients [27]. A study conducted in the USA reported a prevalence of burnout syndrome of 51% among board-certified neurosurgeons and 45% among neurosurgical residents [2]. Comparable data from Germany showed that almost 50% of all surgeons met the criteria for burnout [28]. Moreover, 55% of attending orthopedic surgeons and 40% of trainees screened positive for burnout according to a Canadian survey [29].

We found a statistically significant correlation between high perceived stress, mental burden and lower quality of life. According to a multivariate analysis, self-reported resilience, perceived stress, mental composite score of the SF-8, and job category (senior vs. resident physician) contributed to significant burden. Previous studies have reported similar findings, such as working hours and trauma surgery in a clinical setting, and younger age, large amount of non-clinical activities, incentive pay, and working in a private setting as promoting lower quality of life [30]. Interestingly, physicians who engage in multitasking activities tend to self-report better performance but at the cost of increased psychophysical strain [31], suggesting that efficient task allocation may reduce work-related stress.

### High resilience is an important protective factor against mental burden

The quality of life of spine surgeons was generally similar to that of the general population in all domains. It is possible that the high-level of work-related stress can be compensated by high resilience and therefore does not translate into lower quality of life. The level of work ability in surgeons was high despite the highest level of perceived burden among medical specialties [10]. This idea is supported by a significant association between perceived stress and GHQ scores, as well as BRS and MCS in regression analysis.

In our study, the level of resilience was significantly higher among spine surgeons than among the general population. Self-efficacy, defined as the belief in one's own capabilities is a significant factor in overcoming stress in a variety of situations. High levels of self-efficacy and personal accomplishment among surgical residents have been associated with their well-being [32] and inversely associated with burnout [33]. Rodriguez-Unda et al. also reported a protective effect of resilience a burnout [34].No differences in self-efficacy were found between specialties or number of postgraduate years [32].

### Work experience leads to lower mental burden

Chairpersons and vice-chairpersons demonstrated the highest levels of resilience and self- efficacy in our study, suggesting that either individuals develop protective skills during their careers or that those individuals with the highest values are the most successful in their careers. Residents appear to be the most vulnerable professional group according to our data, reporting the highest levels of burden and the lowest resilience. Younger, less experienced physicians are particularly vulnerable to mental burden [3, 35]. These findings are consistent with similar studies, showing that physicians with significantly fewer years of practice were classified as distressed [29]. However, the most significant factors for psychological burden are workload, followed by poor professional relationships, work–home conflicts, and poor work–life-balance [3, 35].

Protective factors against work-related stress include marriage or spousal support, career satisfaction, autonomy, and academic practice [35]. On the other hand, stress during residency can be viewed as a facilitator of a personal and professional growth. Exposure to appropriately supervised stress and pressure in a “healthy” surgical environment is an important component of surgical training [36]. Furthermore, there was a negative correlation between increasing number of operations and perceived stress. In general, surgical residents have higher resilience than medical residents [37]. As it appears that with growing experience the level of burden is decreasing, spine surgeons in training are the most exposed to work-related stress. Reducing work hours and maintaining the number of surgeries to develop specialists who meet quality standards is a difficult equation to solve. Therefore, the development of resilience skills as part of the residency program or informal training may be an attractive alternative that needs to be discussed in a formal setting.

#### Reducing occupational stress to prevent psychological problems

A variety of short- and long-term interventions have been proposed and studied to reduce work-related stress and its negative consequences in healthcare workers. A recent systematic review by the Cochrane Collaboration classified the interventions into four categories, depending on whether they focused attention on or away from the experience of stress, whether they tried to change work-related risk factors, or whether they combined two or more of the above. The review included 86 trials and found a short-term effect, regardless of the type of intervention. Although long-term effects remain unknown, there is good evidence that recognising and addressing the problem and offering preventive interventions is effective [38].

## Strength and Limitations

This study has two limitations. The participation rate cannot be accurately determined due to the anonymous nature of the survey and the fact that many spine surgeons are members of both the DWG and the DGNC.

The second limitation is the under-representation of residents and less experienced surgeons in our survey, which may be due to the fact that more experienced surgeons tend to be more active in professional societies.

The strengths of this study include the relatively high number of respondents, the comprehensive nature of the multi-questionnaire survey and the interdisciplinary analysis.

## Conclusion

Spine surgeons are exposed to higher levels of stress than the general population, which are associated with higher mental distress, especially at the beginning of their careers. More professional experience and higher levels of psychological resilience are associated with lower levels of stress.

### Supplementary Information

Below is the link to the electronic supplementary material.

Supplementary file1 (PDF 90 KB)

## Data Availability

All of the material is owned by the authors and no permissions are required.

## References

[1] Balch CM, Shanafelt TD, Dyrbye L (2010). Surgeon distress as calibrated by hours worked and nights on call. J Am Coll Surg Published Online First.

[2] Zaed I, Jaaiddane Y, Chibbaro S (2020). Burnout Among Neurosurgeons and Residents in Neurosurgery: A Systematic Review and Meta-Analysis of the Literature. World Neurosurg.

[3] Dimou FM, Eckelbarger D, Riall TS (2016). Surgeon burnout: A systematic review. J Am Coll Surg.

[4] West CP, Dyrbye LN, Sinsky C (2020). Resilience and Burnout Among Physicians and the General US Working Population. JAMA Netw open.

[5] Dutheil F, Aubert C, Pereira B (2019). Suicide among physicians and health-care workers: A systematic review and meta-analysis. PLoS One.

[6] Elkbuli A, Sutherland M, Shepherd A, et al (2020) Factors Influencing US Physician and Surgeon Suicide Rates 2003–2017: Analysis of the CDC-National Violent Death Reporting System. Ann Surg Published Online First: 4. 10.1097/SLA.0000000000004575 10.1097/SLA.0000000000004575 33156059

[7] Delongis A, Coyne JC, Dakof G, et al (1982) Relationship of Daily Hassles, Uplifts, and Major Life Events to Health Status. https://psycnet.apa.org/record/1983-05622-001 (accessed 14 Feb 2021)

[8] Chmitorz A, Kunzler A, Helmreich I (2018). Intervention studies to foster resilience – A systematic review and proposal for a resilience framework in future intervention studies. Clin Psychol Rev.

[9] Pulcrano M, Evans SRT, Sosin M (2016). Quality of life and burnout rates across surgical specialties: A systematic review. JAMA Surg.

[10] Bernburg M, Vitzthum K, Groneberg DA, et al (2016) Physicians’ occupational stress, depressive symptoms and work ability in relation to their working environment: A cross-sectional study of differences among medical residents with various specialties working in German hospitals. BMJ Open 6. 10.1136/bmjopen-2016-011369 10.1136/bmjopen-2016-011369 PMC4916614 27311909

[11] Turner-Bowker DM, Bayliss MS, Ware JE (2003). Usefulness of the SF-8™ Health Survey for comparing the impact of migraine and other conditions. Qual Life Res.

[12] Maruish ME, DeRosa MA. (2009) A Guide to the Integration of Certified Short Form Survey Scoring and Data Quality Evaluation Capabilities

[13] Kalliath TJ, O’Driscoll MP, Brough P (2004). A confirmatory factor analysis of the General Health Questionnaire - 12. Stress Heal.

[14] Schmitz N, Kruse J, Tress W (1999). Psychometric properties of the General Health Questionnaire (GHQ-12) in a German primary care sample. Acta Psychiatr Scand.

[15] Cohen S, Kamarck T, Mermelstein R (1994). Perceived stress scale. Meas Stress A Guid Heal Soc Sci.

[16] Engling J GfK SE (2010) Fragen zum Thema Stress. PSS-4 Deutsch, [Questions relating to stress: German PSS-4]. http://www.psy.cmu.edu//~scohen//scales.html. 2010. http://www.psy.cmu.edu//~scohen//scales.html

[17] Chmitorz A, Wenzel M, Stieglitz R-D (2018). Population-based validation of a German version of the Brief Resilience Scale. PLoS One.

[18] Kunzler AM, Chmitorz A, Bagusat C (2018). Construct Validity and Population-Based Norms of the German Brief Resilience Scale (BRS). Eur J Heal Psychol.

[19] Smith BW, Dalen J, Wiggins K (2008). The brief resilience scale: Assessing the ability to bounce back. Int J Behav Med.

[20] Bandura A (1977). Self-efficacy: Toward a unifying theory of behavioral change. Psychol Rev.

[21] MacHe S, Vitzthum K, Wanke E (2014). Exploring the impact of resilience, self-efficacy, optimism and organizational resources on work engagement. Work.

[22] Beierlein C, Kovaleva A, Kemper CJ, et al. (2014) Allgemeine Selbstwirksamkeit Kurzskala (ASKU). Zusammenstellung sozialwissenschaftlicher Items und Skalen

[23] Romppel M, Braehler E, Roth M (2013). What is the General Health Questionnaire-12 assessing?: Dimensionality and psychometric properties of the General Health Questionnaire-12 in a large scale German population sample. Compr Psychiatry.

[24] Gadjradj PS, Ghobrial JB, Booi SA, de Rooij JD, Harhangi BS (2021). Mistreatment, discrimination and burn-out in Neurosurgery. Clin Neurol Neurosurg.

[25] Mahoney ST, Irish W, Strassle PD (2020). Practice Characteristics and Job Satisfaction of Private Practice and Academic Surgeons. JAMA Surg Published Online First.

[26] Janus K, Amelung VE, Baker LC (2008). Job satisfaction and motivation among physicians in academic medical centers: Insights from a cross-national study. J Health Polit Policy Law.

[27] Klein J, Frie KG, Blum K, et al. (2011) Psychosocial stress at work and perceived quality of care among clinicians in surgery. BMC Health Serv Res 11. 10.1186/1472-6963-11-109 10.1186/1472-6963-11-109 PMC3119178 21599882

[28] Klein J, Grosse Frie K, Blum K (2010). Burnout and perceived quality of care among German clinicians in surgery. Int J Qual Heal Care.

[29] Kollias CM, Okoro T, Tufescu TV (2020). Distress in orthopedic trainees and attending surgeons: A Canadian national survey. Can J Surg.

[30] Balch CM, Shanafelt TD, Sloan JA (2011). Distress and Career Satisfaction Among 14 Surgical Specialties, Comparing Academic and Private Practice Settings. Ann Surg.

[31] Weigl M, Müller A, Sevdalis N (2013). Relationships of multitasking, physicians’ strain, and performance: An observational study in ward physicians. J Patient Saf.

[32] Milam LA, Cohen GL, Mueller C (2019). The Relationship Between Self-Efficacy and Well-Being Among Surgical Residents. J Surg Educ.

[33] Janko MR, Smeds MR (2019). Burnout, depression, perceived stress, and self-efficacy in vascular surgery trainees. J Vasc Surg.

[34] Rodriguez-Unda NA, Mehta I, Chopra S, Vicente-Ruiz M, Navia A, Fernandez-Diaz OF (2023). Global Resilience in Plastic Surgery Study (GRIPS): Resilience is Associated with Lower Burnout Rates. Plast Reconstr Surg Glob Open.

[35] Oskrochi Y, Maruthappu M, Henriksson M (2016). Beyond the body: A systematic review of the nonphysical effects of a surgical career. Surg Surg (United States).

[36] Spiotta AM (2019). Pursuing Wellness in Neurosurgery: Resiliency. Clin Neurosurg.

[37] Nituica C, Bota OA, Blebea J (2021) Specialty differences in resident resilience and burnout - A national survey. Am J Surg Published Online First: 25 December 2020. 10.1016/j.amjsurg.2020.12.039 10.1016/j.amjsurg.2020.12.039 33431168

[38] Tamminga SJ, Emal LM, Boschman JS (2023). Individual-level interventions for reducing occupational stress in healthcare workers. Cochrane Database Syst Rev.

