# Supplementary material for: How do spine surgeons cope with psychological distress: results of a cross-sectional study
Source: Neurosurg Rev. 2023 Jul 22;46(1):182. doi: 10.1007/s10143-023-02088-z (PMC10363079; doi:10.1007/s10143-023-02088-z)
Supplement: Supplementary file 1 — Supplementary file1 (PDF 90 KB) [file 10143_2023_2088_MOESM1_ESM.pdf]

* 1. Wie alt sind Sie? (Jahre)

[ ]

* 2. Sind Sie weiblich oder männlich?

- ◯ Weiblich
- ◯ Männlich

* 3. Ihr aktueller Familienstand:

- ◯ verheiratet/eingetragene Lebenspartnerschaft/feste Partnerschaft
- ◯ geschieden/eingetragene Lebenspartnerschaft aufgehoben/getrennt lebend
- ◯ ledig
- ◯ verwitwet

* 4. Wie viele Kinder haben Sie?

[ ]

* 5. Leben Ihre Kinder in Ihrem Haushalt?

- ◯ ja
- ◯ nein
- ◯ ich habe keine Kinder

* 6. Wo arbeiten Sie?

- ◯ Universitätsklinik
- ◯ sonstiges Krankenhaus
- ◯ ambulante Praxis
- ◯ Industrie
- ◯ Sonstiges (bitte angeben)

* 7. Was ist Ihre Fachrichtung?

- ◯ Unfallchirurgie / Orthopädie
- ◯ Neurochirurgie
- ◯ Sonstiges (bitte angeben)

* 8. Haben Sie die Prüfung zum Facharzt abgelegt?

- ◯ Facharztprüfung noch nicht absolviert
- ◯ Falls Sie die Facharztprüfung absolviert haben, bitte das Jahr angeben (JJJJ)

* 9. Was ist Ihre Berufsgruppe/bezeichnung?

- ◯ Chefarzt
- ◯ Funktions-Oberarzt

- ◯ leitender Oberarzt
- ◯ Oberarzt
- ◯ Assistenzarzt
  Jahr der Weiterbildung :
- ◯ Facharzt

* 10. Wie viele Operationen führen Sie selbst im Jahr durch?

- ◯ <50
- ◯ 50-100
- ◯ 100-200
- ◯ 200-400
- ◯ 400-800
- ◯ >800

* 11. Wie viele Stunden arbeiten Sie durchschnittlich pro Woche (incl. Dienste) ? _____ Std/Woche

* 12. Wie hoch war Ihr Bruttoverdienst (in €, vor Abzug der Lohnnebenkosten) im vergangenen Jahr (inkl. aller Zusatzzahlungen)?

- ◯ <40.000€
- ◯ ≥40.000 bis <100.000€
- ◯ ≥ 100.000 bis <150.000€
- ◯ ≥ 150.000 bis < 200.000€
- ◯ ≥ 200.000€

* 13. Im welchem Land arbeiten Sie?

◯ Deutschland

◯ Schweiz

◯ Österreich

◯ Sonstiges (bitte angeben)

[ ]

* 14. Sind Sie selbst mit Ihrem beruflichen Erfolg zufrieden?

◯ ja

◯ nein

* 15. Wie gut beurteilen Sie Ihre Aufstiegschancen?

◯ sehr gut ◯ gut ◯ mäßig

◯ eher schlecht ◯ schlecht

* 16. Wurde bei Ihnen jemals eine der folgenden psychischen Erkrankungen diagnostiziert?

◯ Nein

◯ ADHS/ADHD/ADS

◯ Depression

◯ Angststörung

◯ Sonstige psychische Erkrankung, und zwar :

* 17. Wie oft trinken Sie Alkohol?

- ◯ nie
- ◯ etwa ein mal im Monat
- ◯ zwei bis vier mal im Monat
- ◯ zwei bis vier mal in der Woche
- ◯ fünf mal oder mehr in der Woche

18. Wenn Sie an einem Tag Alkohol trinken, wir viele alkoholhaltige Getränke (in Gläsern) trinken Sie dann typischerweise?

◯ 1-2 ◯ 3-4 ◯ 5-6 ◯ 7-9

◯ 10 oder mehr

* 19. Wie viele Zigaretten rauchen Sie im Allgemeinen pro Tag?

- ◯ Ich rauche nicht
- ◯ Ja.
  Anzahl der Zigaretten pro Tag :

* 20. Seit wie vielen Jahren rauchen Sie?

- ◯ Ich rauche nicht
- ◯ Anzahl der Jahre :

* 21. Wieviele der von Ihnen durchgeführten Eingriffe sind Wirbelsäulenoperationen?

- ◯ 0%
- ◯ 1-25%
- ◯ 25%-50%
- ◯ 50%-75%
- ◯ 75-99%
- ◯ 100%
